# Identification of new type I interferon-stimulated genes and investigation of their involvement in IFN-β activation

**DOI:** 10.1007/s13238-018-0511-1

**Published:** 2018-02-09

**Authors:** Xiaolin Zhang, Wei Yang, Xinlu Wang, Xuyuan Zhang, Huabin Tian, Hongyu Deng, Liguo Zhang, Guangxia Gao

**Affiliations:** 1 CAS Key Laboratory of Infection and Immunity, CAS Center for Excellence in Biomacromolecules, Institute of Biophysics, Chinese Academy of Sciences, Beijing, 100101 China; 2 University of Chinese Academy of Sciences, Beijing, 100049 China

**Keywords:** interferon-stimulated genes, IFN-β signaling, PIM1, RIG-I, MDA5

## Abstract

Virus infection induces the production of type I interferons (IFNs). IFNs bind to their heterodimeric receptors to initiate downstream cascade of signaling, leading to the up-regulation of interferon-stimulated genes (ISGs). ISGs play very important roles in innate immunity through a variety of mechanisms. Although hundreds of ISGs have been identified, it is commonly recognized that more ISGs await to be discovered. The aim of this study was to identify new ISGs and to probe their roles in regulating virus-induced type I IFN production. We used consensus interferon (Con-IFN), an artificial alpha IFN that was shown to be more potent than naturally existing type I IFN, to treat three human immune cell lines, CEM, U937 and Daudi cells. Microarray analysis was employed to identify those genes whose expressions were up-regulated. Six hundred and seventeen genes were up-regulated more than 3-fold. Out of these 617 genes, 138 were not previously reported as ISGs and thus were further pursued. Validation of these 138 genes using quantitative reverse transcription PCR (qRT-PCR) confirmed 91 genes. We screened 89 genes for those involved in Sendai virus (SeV)-induced IFN-β promoter activation, and PIM1 was identified as one whose expression inhibited SeV-mediated IFN-β activation. We provide evidence indicating that PIM1 specifically inhibits RIG-I- and MDA5-mediated IFN-β signaling. Our results expand the ISG library and identify PIM1 as an ISG that participates in the regulation of virus-induced type I interferon production.

**Electronic supplementary material:**

The online version of this article (10.1007/s13238-018-0511-1) contains supplementary material, which is available to authorized users.

## Introduction

Viral infection activates host innate immune response (Schneider et al., 2014). The retinoic acid-inducible gene I (RIG-I)-like receptors (RLRs), RIG-I and melanoma differentiation-associated gene 5 (MDA5), are important to initiate innate immune in response to RNA virus invasion (Wilkins and Gale, 2010). Following recognition of viral RNAs, RLRs are recruited to an adaptor protein VISA (also known as MAVS, IPS-1 and Cardif), which further triggers TBK1/IKKε and IKKα/β kinases-mediated activation of IRF3 and NF-κB (Xu et al., 2005; Seth et al., 2005; Meylan et al., 2005; Kawai et al., 2005). These events ultimately lead to the induction of the expressions of type I IFNs and pro-inflammatory cytokines.

Interferons (IFNs) are a group of pleiotropic cytokines that are made and released by host cells in response to pathogen infections and tumorgenesis (Pestka, 2007). Based on their receptors, IFNs are divided into three classes (Uze et al., 2007; de Weerd et al., 2007). Type I IFNs comprise IFN-α, IFN-β, IFN-ε, IFN-κ and IFN-ω, which bind to the type I IFN heterodimeric receptor complex of IFN-α receptor 1 (IFNAR1) and receptor 2 (IFNAR2) (Chen et al., 2004). Type II IFN, IFN-γ, signals through the IFN-γ receptor complex (IFNGR) (Pestka et al., 1997) and type III IFNs signal through IFN-λ receptor 1 or IL-10R2 (Kotenko et al., 2003).

Type I IFN binding to IFNAR activates a signaling cascade through the Janus kinase and signal transducer and activator of transcription (JAK-STAT) pathway (Aaronson and Horvath, 2002). This leads to the assembly of the IFN-stimulated gene factor 3 (ISGF3) complex, which is composed of STAT1-STAT2 dimers and IFN-regulatory factor 9 (IRF9) (Fu et al., 1992). The complex translocates to the nucleus and binds to the IFN-stimulated response elements (ISREs) present in the promoters of IFN-stimulated genes (ISGs), thereby initiating the transcription of those genes.

Systematic identification of ISGs revealed that there may be more than a thousand ISGs (Martensen and Justesen, 2004; Schoggins and Rice, 2011). In these studies, the naturally existing IFN-α was commonly used as the stimulator. However, there are 13 IFN-α subtypes in addition to the other type I IFNs. Although these type I IFNs use the same receptor, their downstream effects are different to some extents. Based on the sequence alignment, Alton et al. designed a consensus alpha IFN (Con-IFN) (Alton, 1983). Compared with the naturally existing recombinant type I IFN (IFN-α2a and IFN-α2b), Con-IFN displayed remarkable enhanced natural killer cell activation, antiviral, antiproliferative, and gene-induction activities (Klein et al., 1988; Blatt et al., 1996). Here we used Con-IFN to stimulate three immune cell lines and identified dozens of new ISGs.

Although innate immune responses provide one of the first lines of defense against viral infection (Schneider et al., 2014), uncontrolled immune activation could be harmful to the host (Yap and Lai, 2010); the IFN signaling needs to be tightly controlled. A number of ISGs have been reported to play roles in the maintenance of the homeostasis. For example, several components in the RLR pathway are IFN responsive, such as RIG-I, IRF3 and IRF7 (Schneider et al., 2014; Schoggins et al., 2011). The induction of these proteins in turn reinforces IFN production. The expression of some TRIM proteins, such as TRIM13 and TRIM25, is up-regulated by type I IFNs (Rajsbaum et al., 2008). These proteins also play important roles in the regulation of immune responses (Narayan et al., 2014; Gack et al., 2007; Versteeg et al., 2013).

We screened the newly identified ISGs for those participating in the modulation of virus-induced innate immune activation. PIM1 was found to negatively regulate Sendai virus (SeV)-triggered IFN-β promoter activation.

## Results

### Identification of ISG candidates by microarray analysis

**Figure 1:**
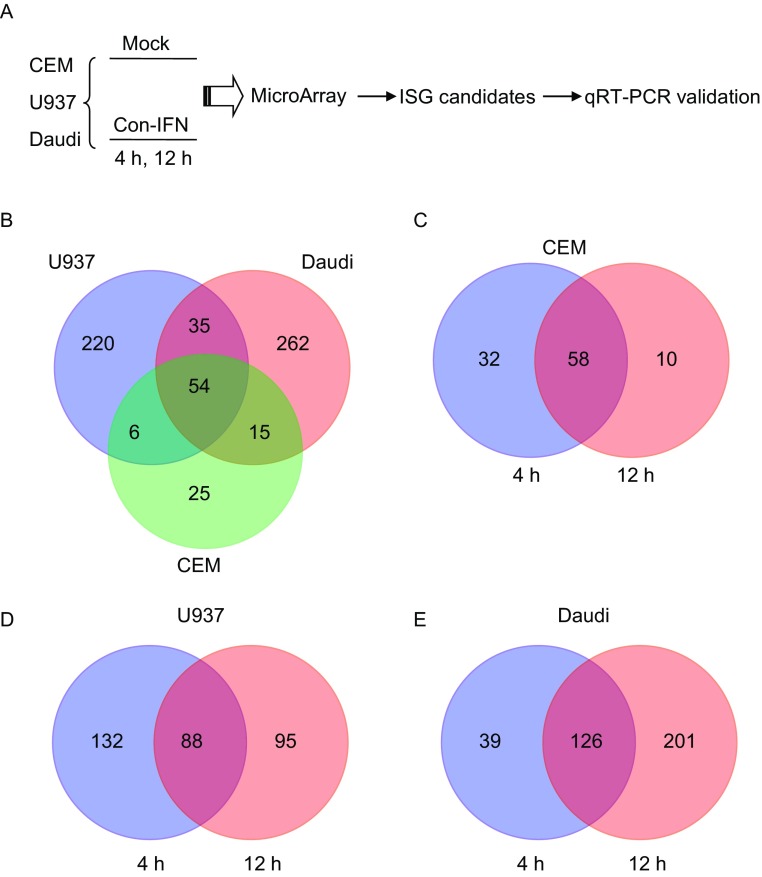
**Identification of ISG candidates by microarray analysis**. (A) Flowchart of the identification of ISG candidates. CEM, U937 and Daudi cells were mock treated, or treated with Con-IFN for 4 h or 12 h. The RNAs were isolated and reverse transcribed into cDNAs, followed by microarray analyses. Those up-regulated more than 3-fold in any cell lines with either 4 h or 12 h treatment were considered as ISG candidates and subjected to validation by qRT-PCR. (B) ISG candidates specific and common in CEM, U937 and Daudi cells were categorized and represented in a Venn diagram. (C–E) ISG candidates in CEM (C), U937 (D) and Daudi cells (E) that were up-regulated more than 3-fold upon treatment with Con-IFN for 4 h or 12 h were categorized and represented in Venn diagrams. Data are from two parallel experiments, *P* < 0.05

To identify ISG candidates, we used Con-IFN to treat three human immune cell lines, CD4^+^ T-cell-derived CEM, monocyte-derived U937 and B cell-derived Daudi, for 4 or 12 h (Fig. 1A). The mRNA expression profiles were analyzed using microarrays that covered 29,185 genes. The mRNA expression patterns in different cell lines were different to some extents (Fig. 1B). In the same cell line, the mRNA expression patterns with Con-IFN treatment for different periods of time were also different (Fig. 1C–E). Those genes whose mRNA levels were up-regulated more than 3-fold upon Con-IFN treatment for either 4 h or 12 h in any cell type were considered as ISG candidates. By this criterion, 617 genes were considered as ISG candidates, all with corrected *P* values <0.05. Since the aim of this study was to identify new ISGs, we searched the literature to exclude those that had been previously reported. Considering that most ISGs were identified in the research for antiviral innate immunity, we searched the literature using the above gene symbol and virus as keywords. Negative results were obtained with 242 genes. Among these 242 genes, 104 had been reported as ISGs by two papers that described systematic identification of ISGs (Schoggins et al., 2011; Liu et al., 2012). We focused on the remaining 138 genes as ISG candidates for further studies.

### Identification of new ISGs by qRT-PCR

**Figure 2:**
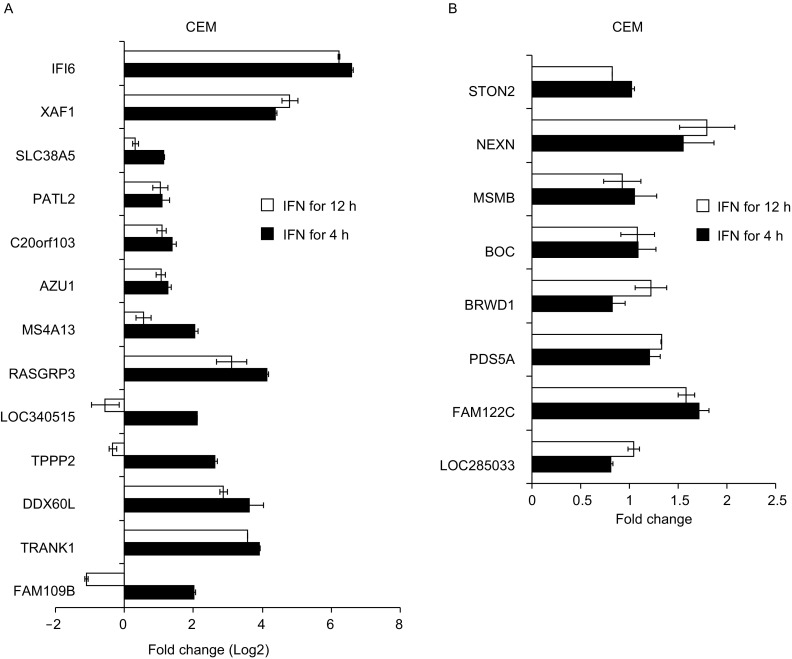
**Validation of new ISG candidates in CEM cells by qRT-PCR**. CEM cells were treated with Con-IFN for 0 h, 4 h or 12 h. The mRNA levels of the ISG candidates were analyzed by qRT-PCR. The ISG candidate mRNA levels were normalized with GAPDH levels. IFI6 and XAF1, two known ISGs, were used as positive controls. Fold change was calculated as the mRNA level in the cells with Con-IFN treatment divided by that without treatment. Data represented are mean ± SD of two independent measurements

**Figure 3:**
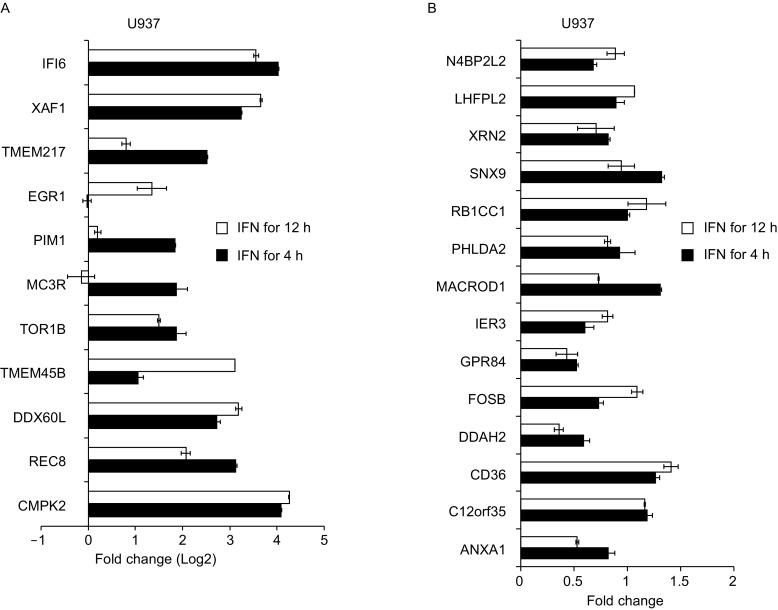
**Validation of new ISG candidates in U937 cells by qRT-PCR**. U937 cells were treated with Con-IFN for 0 h, 4 h or 12 h. The mRNA levels of new ISG candidates were analyzed by qRT-PCR, as described in the legend to Fig. 2. Data represented are mean ± SD of two independent measurements

We used quantitative reverse transcription-PCR (qRT-qPCR) to validate the 138 ISG candidates. Two known ISGs, IFI6 and XAF1 (Schoggins et al., 2011), were used as positive controls. Because the gene expression patterns are different in different cell lines (Fig. 1), we analyzed the mRNA levels of different ISG candidates in different cell lines based on the microarray results. Nineteen candidates in CEM cells (Fig. 2), 23 in U937 cells (Fig. 3) and 97 in Daudi cells (Fig. S1) were analyzed for up-regulation by Con-IFN treatment for 4 h or 12 h. In all the three cell lines, the two positive control genes were up-regulated (Figs. 2A, 3A and S1A), confirming the reliability of the qRT-PCR analysis. Based on these results, 91 genes were confirmed as ISGs (fold induction ≥2) (Fig. S1C), and 41 genes were not confirmed to be ISGs. For the rest six genes, the results were not conclusive, due to either their low expression levels in the cells or nonspecific amplification of the PCR product.

### Screen for the ISGs that modulate SeV-triggered IFN-β activation

**Figure 4:**
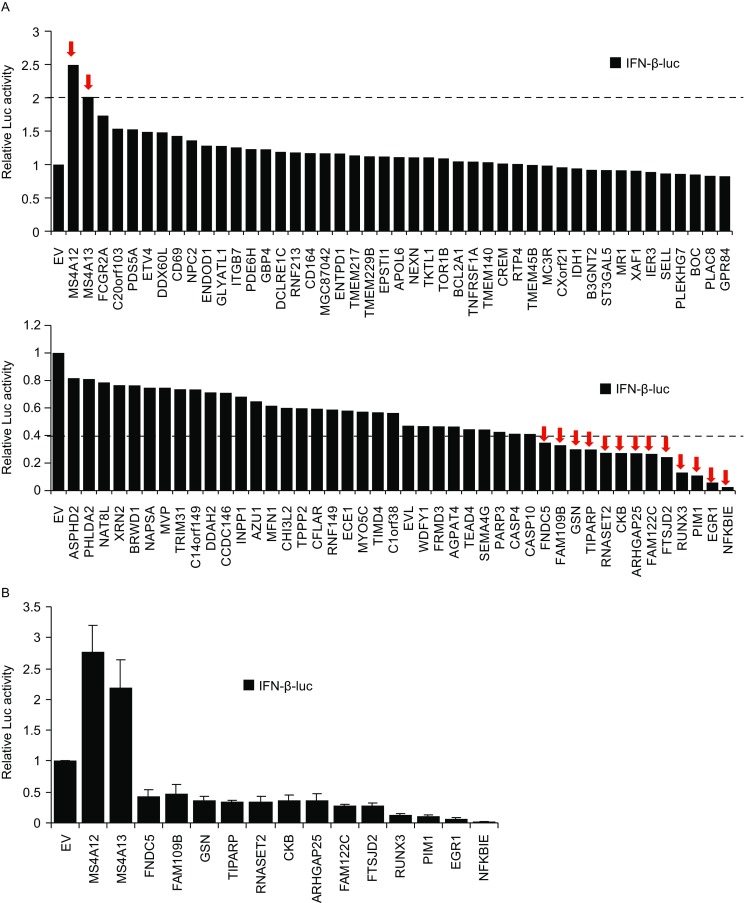
**Screen for ISGs involved in SeV-induced IFN-β promoter activation**. (A) HEK293T cells were transfected with the firefly luciferase-expressing reporter IFN-β-luc and the renilla luciferase-expressing control reporter TK-renilla, with or without a plasmid expressing the ISG indicated. At 24 h post-transfection, cells were infected with 10 HAU/mL SeV for 12 h. The cells were then lysed and luciferase activities were measured. Firefly luciferase activity was normalized with renilla luciferase activity. The relative luciferase activity in the empty vector-transfected cells was set as 1. (B) The experiment was repeated with those ISGs indicated by red arrows in (A). Data represented are mean ± SD of three independent experiments

We next screened the new ISGs for their involvement in regulating virus-induced activation of innate immunity. HEK293T cells were transfected with a firefly luciferase reporter under the control of the IFN-β promoter (IFN-β-luc), with or without a plasmid expressing the ISG. The cells were then challenged with SeV. SeV infection significantly activated the expression of the IFN-β-luc reporter (Fig. S2). The effect of the ISG on SeV-triggered IFN-β-luc expression was indicated by the fold change, calculated as the luciferase activity with ISG divided by that without ISG. The primary screen compiled a list of 89 new ISGs (Fig. 4A). Two ISGs promoted SeV-triggered IFN-β-luc expression by more than 2-fold and 13 ISGs reduced the SeV-triggered IFN-β promoter activation. The effects of these 15 ISGs were confirmed by repeated experiments (Fig. 4B). These results suggest that ISGs can both positively and negatively regulate the virus-induced IFN production pathway.

### PIM1 negatively regulates SeV-triggered activation of IFN-β promoter

**Figure 5:**
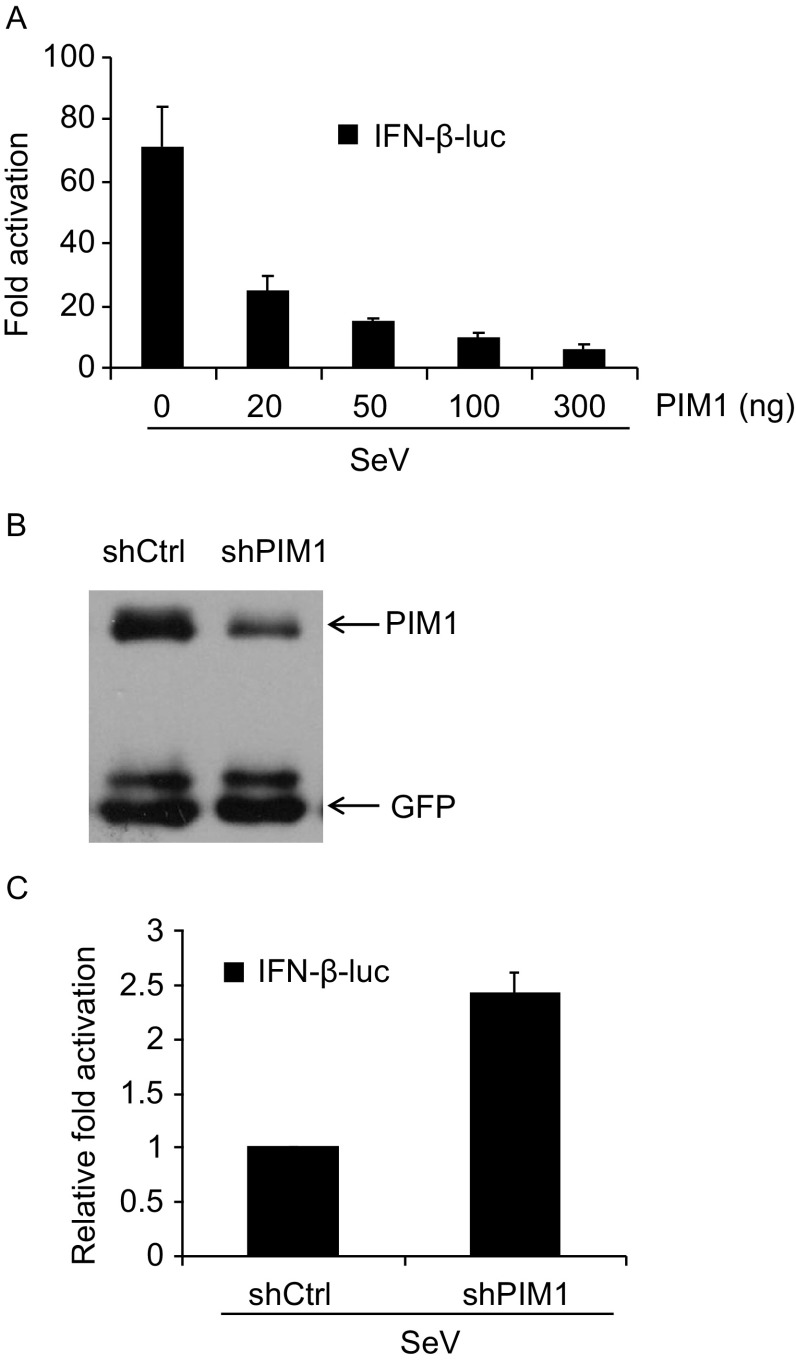
**PIM1 negatively regulates SeV-induced IFN-β promoter activation**. (A) HEK293T cells were transfected with increasing amounts of a plasmid expressing PIM1, together with the luciferase reporters IFN-β-luc and TK-Renilla. The cells were infected with 20 HAU/mL SeV and luciferase activities were measured as in the legend to Fig. 4A. Firefly luciferase activity was normalized with renilla luciferase activity. The fold activation was calculated as the luciferase activity in cells infected with SeV divided by that without SeV. (B) HEK293T cells were transfected with plasmids expressing myc-tagged PIM1 and GFP, together with a plasmid expressing a control shRNA or an shRNA targeting PIM1. At 48 h post-transfection, cells were lysed and subjected to Western blot analyses. (C) HEK293T cells were transfected with the luciferase reporters IFN-β-luc and TK-renilla, together with a plasmid expressing a control shRNA or the shRNA targeting PIM1. At 36 h post-transfection, cells were infected with 20 HAU/mL SeV for 12 h. The cells were then lysed and luciferase activities were measured. Fold activation was calculated as in the legend to panel A. The relative fold activation in the cells transfected with the plasmid expressing the control shRNA was set as 1. Data represented are mean ± SD of two independent experiments

The above screen identified four ISGs whose overexpression inhibited SeV-triggered IFN-β-luc expression by about 10-fold, including NFKBIE, EGR1, PIM1 and RUNX3. As NFKBIE is a known factor negatively regulating the NF-κB pathway, it was not further pursued. PIM1 is a serine/threonine kinase that regulates cell proliferation and survival (Bachmann and Moroy, 2005). We focused on this ISG for further investigation. In the reporter assays, we found that overexpression of PIM1 inhibited SeV-induced IFN-β-luc activation in a dose-dependent manner (Fig. 5A). We constructed an shRNA to downregulate PIM1 expression (Fig. 5B) and examined the effect of PIM1 knockdown on SeV-induced IFN-β promoter activation. Data showed that downregulation of endogenous PIM1 expression promoted SeV-induced IFN-β promoter activation by about 2-fold (Fig. 5C). Collectively, these results indicate that PIM1 negatively regulates SeV-induced IFN-β activation.

### PIM1 inhibits RIG-I- and MDA5-mediated activation of IFN-β promoter

**Figure 6:**
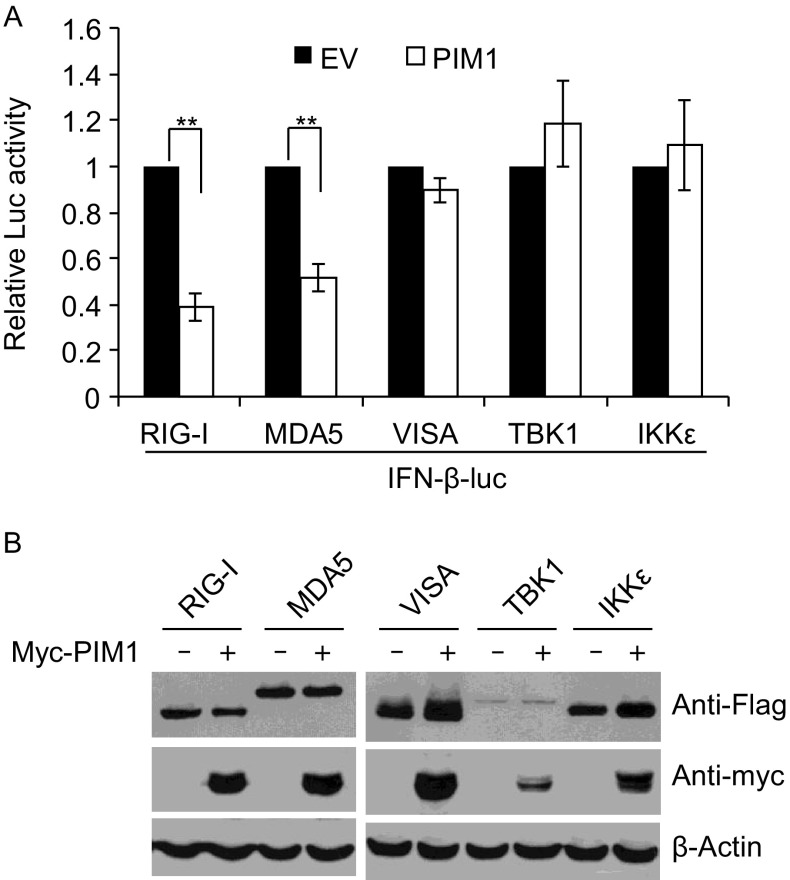
**PIM1 specifically inhibits RIG-I- and MDA5-mediated activation of IFN-β promoter**. HEK293T cells were transfected with the luciferase reporters IFN-β-luc and TK-renilla, together with a plasmid expressing Flag-tagged signal transducer protein indicated and a plasmid expressing myc-tagged PIM1. At 30 h post-transfection, the cells were lysed. (A) Luciferase activities were measured and firefly luciferase activity was normalized with renilla luciferase activity. The relative luciferase activity in the cells without PIM1 was set as 1. Data represented are mean ± SD of three independent experiments. The *P* value is determined by two-tailed Student’s *t* test. **P < 0.01. (B) Cell lysates were analyzed by Western blot

SeV-induced IFN-β activation involves multiple sensors and signal transducers. The viral RNA is detected by RIG-I or MDA5 (Diao et al., 2007), which subsequently activates VISA, TBK1 and IKKε. To define at which step PIM1 inhibits SeV-induced IFN-β activation, we assayed the effect of PIM1 overexpression on the sensor- or signal transducer-induced IFN-β activation. HEK293T cells were transfected with the IFN-β-luc reporter and a plasmid expressing RIG-I, MDA5, VISA, TBK1 or IKKε, with or without a plasmid expressing PIM1. The effect of PIM1 was evaluated based on the ratio of the luciferase activity in the presence of PIM1 to that in the absence of PIM1. Data showed that PIM1 inhibited RIG-I- and MDA5-mediated activation of IFN-β reporter (Fig. 6A). However, PIM1 had little effect on the reporter activation by the signal transducers VISA, TBK1 and IKKε (Fig. 6A). Noticeably, PIM1 did not reduce the protein levels of RIG-I or MDA5 (Fig. 6B). Taken together, these results suggest that PIM1 inhibits RIG-I- and MDA5- activated IFN-β up-regulation, but acts upstream of VISA and TBK1.

## Discussion

Given their important roles in the innate immune defenses, ISGs have been extensively studied. The effects of type I IFNs on the transcriptome of several cell types have been investigated in previous studies (Liu et al., 2012; Der et al., 1998; de Veer et al., 2001; Hilkens et al., 2003; Calcaterra et al., 2006; Indraccolo et al., 2007), and the effects of IFNs on B cells were only partially studied (Salamon et al., 2012; Pfeffer et al., 1991). Unlike previous studies, here we used Con-IFN, a bio-optimized highly potent type I interferon alpha (Blatt et al., 1996), to treat three human immune cell types, T lymphoblast-derived CEM, B lymphoblast-derived Daudi and monocyte-derived U937 cells. The three cell types displayed different gene expression profiles in response to Con-IFN (Fig. 1B). In addition, even for a particular cell type, some genes were induced upon Con-IFN treatment for 4 h but not for 12 h and vice versa (Fig. 1). Our strategy used here helped to increase the coverage of ISGs.

The IFN signaling mediated by RLRs functions as an effective mechanism against RNA virus infection. A few ISGs have been reported to modulate this pathway. We thus tested whether the ISGs identified here were involved in the regulation of the SeV-triggered IFN-β activation. Among the 89 genes tested, 15 affected the pathway (Fig. 4). Only two genes, MS4A12 and MS4A13, enhanced the SeV-triggered IFN-β activation, while the other 13 genes negatively regulated the activation (Fig. 4). These results further suggest that the immune response to viral infection is regulated by multiple mechanisms. Whether these ISGs directly regulate the IFN-β activation or indirectly by interfering with the viral replication needs to be further investigated.

Among the above 13 ISGs that negatively regulated the SeV-triggered IFN activation, the serine/threonine kinase PIM1 displayed strong inhibitory activity (Fig. 4). We focused on this protein for further investigation for two reasons. First, PIM1 is a phosphokinase and phosphorylation of components in the RLR pathways is a common mechanism to modulate the pathway (Bachmann and Moroy, 2005; Quicke et al., 2017; Ivashkiv and Donlin, 2014). Second, it has been reported that inhibition of PIM1 with an inhibitor suppressed viral infection, postulating the possibility that PIM1 is involved in innate immune response (de Vries et al., 2015). Our results showed that PIM1 inhibited both MDA5- and RIG-I-mediated IFN-β promoter activation (Fig. 6). De-phosphorylation and the following poly-ubiquitination of RIG-I and MDA5 are required for their activation. Upon activation, the RLRs translocate to mitochondria and mitochondria-associated membranes where they interact with VISA, then trigger the downstream signaling (Quicke et al., 2017; Wies et al., 2013). PIM1 may interfere with the conformational changes of RIG-I and MDA5 via phosphorylation and disturb their interactions with the downstream adapter VISA. Protein kinase C-α (PKC-α), PKC-β and casein kinase II (CK2) are responsible for phosphorylation of RIG-I (Maharaj et al., 2012; Sun et al., 2011). However, proteins involved in the phosphorylation of MDA5 were not reported. It would be intriguing to investigate whether PIM1 is the undiscovered kinase to phosphorylate MDA5 in future studies.

In summary, our results here expand the ISG library and provide additional evidence that ISGs can negatively regulate the virus-induced type I IFN production. The antiviral activities of the new ISGs and their biological functions *in vivo* await further investigation.

## Materials and methods

### Cell culture

CEM, U937 and Daudi cells were maintained in RPMI 1640 medium (Invitrogen) supplemented with 10% heat-inactivated (56°C, 30 min) fetal bovine serum (Gibco). HEK293T cells were maintained in DMEM supplemented with 10% fetal bovine serum (Gibco).

### Plasmids and viruses

Plasmids expressing ISGs were cloned by standard molecular biology techniques. IFN-β-luc reporter, pTK-renilla, Flag-tagged RIG-I, MDA5, VISA, TBK1 and IKKε were generous gifts from Dr. Hongbing Shu (Wuhan University, China). The plasmids expressing shRNAs were generated by annealing pairs of oligonucleotides and cloning into pSuper-Retro (OligoEngine). The target sequences are as follows: Ctrli: 5′-GCGCGCTTTGTAGGATTCG-3′; shPIM1: 5′-CCATCCATGGATGCAAGAT-3′. SeV was kindly provided by Zhengfan Jiang (Peking University, China).

### Microarray analysis

CEM, U937 and Daudi cells were treated with consensus interferon (1000 IU/mL; Interferon Alfacon-1; Amgen) for 0 h, 4 h or 12 h. Total RNA was extracted with the TRIzol reagent (Invitrogen, USA) following the manufacturer’s instructions. Whole genome transcript analysis was performed by Phalanx Biotech Group.

### Quantitative reverse transcription-PCR

Total RNA was reverse transcribed using random primer in a 20 μL reaction mixture. Relative mRNA levels of candidate ISGs were measured by SYBR Green real-time PCR (RealmasterMix; SYBR Green; Tiangen) in Rotor-gene 6000 (Corbett Life Science) using the following program: (i) 95°C 10 min, 1 cycle; (ii) 95°C 15 s; 60°C 30 s; 72°C 30 s, 40 cycles. All data are shown as mean value for at least two independent measurements. GAPDH mRNA levels served as internal control. Primers (Table 1) used for the PCR assays are designed using the Primer-BLAST tool (https://www.ncbi.nlm.nih.gov/tools/primer-blast/) or acquired from Primer Bank (https://pga.mgh.harvard.edu/primerbank/). Amplification efficiency was assessed for all primer sets, and primers with efficiencies 90%–110% were used.

### Luciferase reporter assays

HEK293T cells were transfected with reporters IFN-β-luc and pTK-renilla, together with a plasmid expressing an ISG using Neofectin (NeoBiolab) for 24 h, or with an shRNA-expressing plasmid for 36 h. The cells were then infected with SeV for additional 12 h. Samples were lysed in passive lysis buffer (Promega). Firefly and renilla luciferase activities were measured using the dual-luciferase reporter assay system (Promega). Firefly luciferase activity was normalized with the renilla luciferase activity.

## Electronic supplementary material

Below is the link to the electronic supplementary material.

Supplementary material 1 (PDF 139 kb)
