# Supplementary material for: Identification of new type I interferon-stimulated genes and investigation of their involvement in IFN-β activation
Source: Protein Cell. 2018 Feb 9;9(9):799–807. doi: 10.1007/s13238-018-0511-1 (PMC6107486; doi:10.1007/s13238-018-0511-1)
Supplement: Supplementary file 1 — Supplementary material 1 (PDF 139 kb) [file 13238_2018_511_MOESM1_ESM.pdf]

Figure S1. Validation of new ISG candidates in Daudi cells by qPCR

(A) and **(B)** Daudi cells were treated with Con-IFN for 0, 4 or 12 h. The mRNA levels of new ISG candidates were analyzed by quantitative RT-PCR, as described in the legend to Fig. 2. For the positive controls IFI6 and XAF1, the blue bar is for 4 h and red for 12 h. **(C)** Summary of the validation results. The pie chart shows the number of confirmed ISGs in three cell lines. ISGs confirmed in more than one cell line are annotated by "cell line / another cell line".

Figure S2. SeV infection significantly activated the expression of the IFN-β-luc reporter.

HEK293T cells were transfected with the firefly luciferase-expressing reporter IFN-β-luc and the renilla luciferase-expressing control reporter TK-Renilla. At 24 h posttransfection, cells were mock treated or infected with 10 HAU/mL SeV for 12 h. The cells were then lysed and luciferase activities were measured. Firefly luciferase activity was normalized with renilla luciferase activity. The relative luciferase activity in the uninfected cells was set as 1. Data represented are mean ± SD of three independent experiments.

A

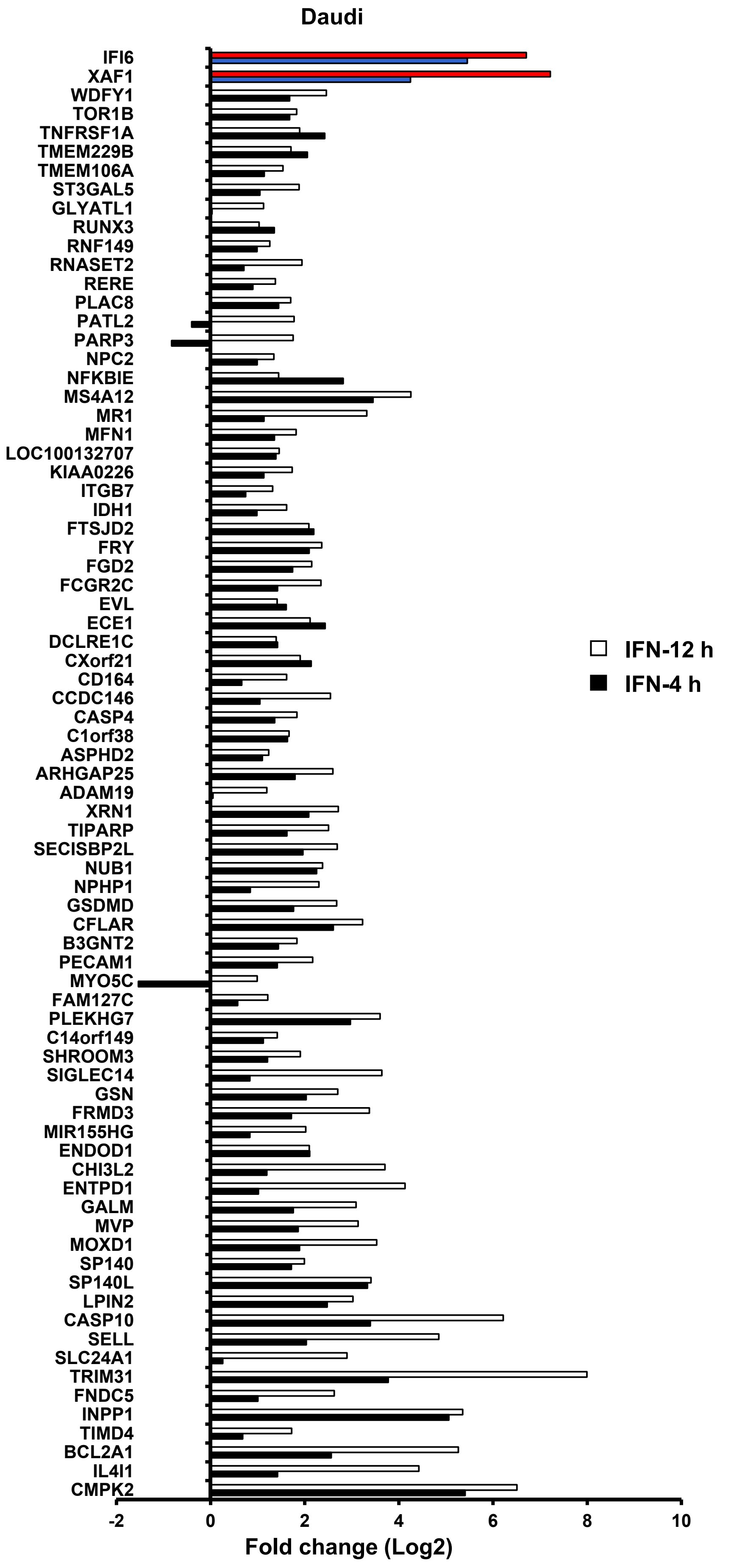

Figure S1. Validation of new ISG candidates in Daudi cells by qPCR

B

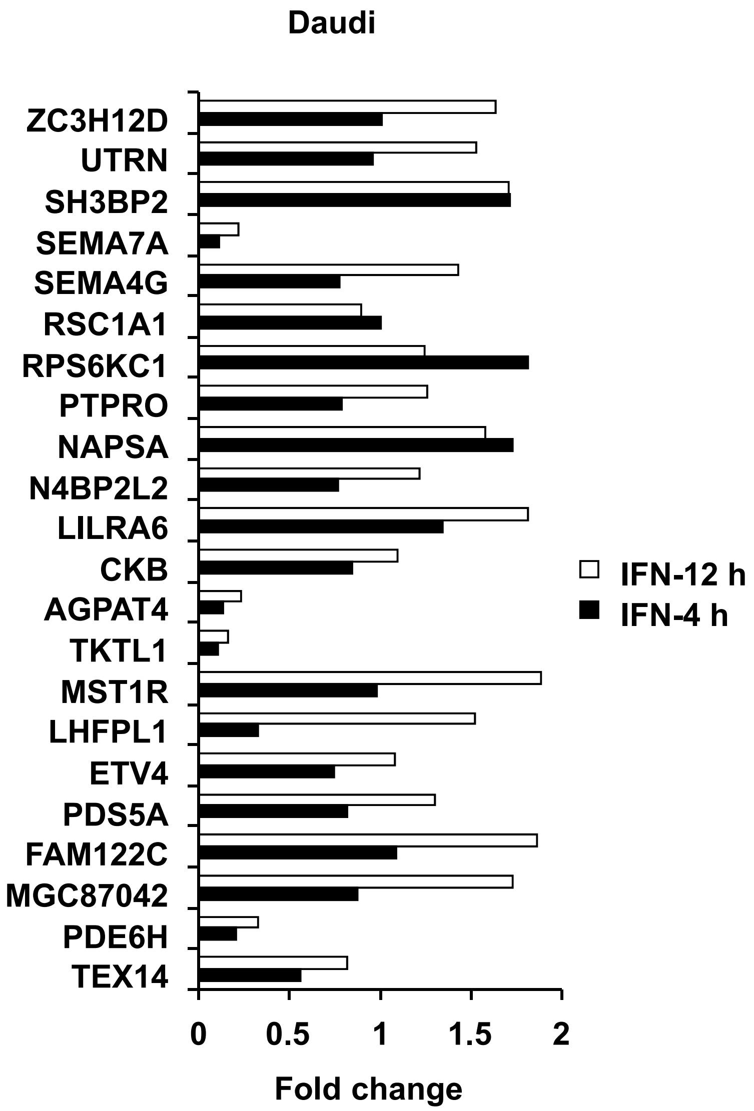

C

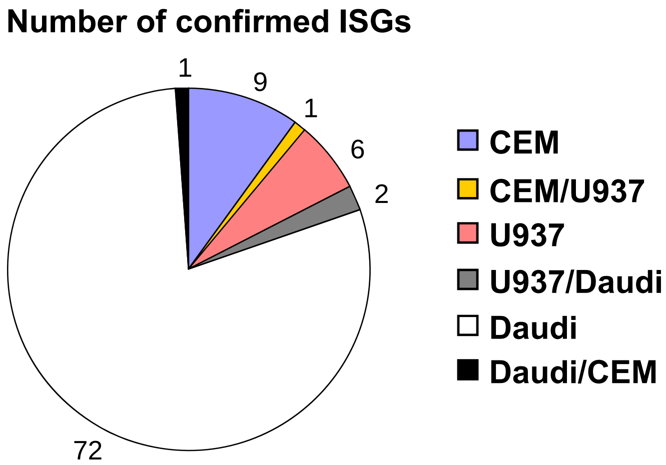

Figure S1. Validation of new ISG candidates in Daudi cells by qPCR

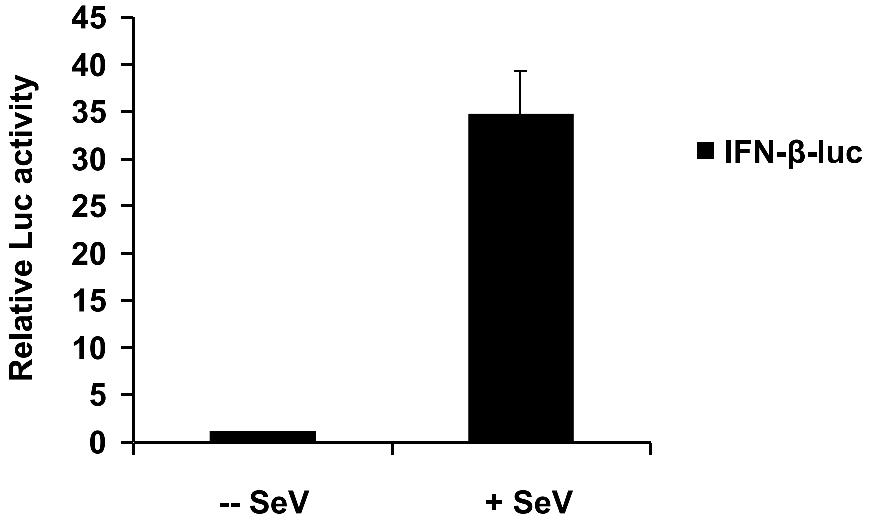

Figure S2. SeV infection significantly activates the expression of the IFN-β-luc reporter

Table 1 qPCR primers

Gene Symbol	Forward Primer(5'-3')	Reverse Primer(5'-3')
ADAM19	GAGGGGCGAGAACTGATCCT	TGGTTTGAGGGTTACCACTTGA
AGPAT4	CTCAGGGCTAATCATCAACACC	GCTTGAGATGCAATAGGACAGT
ANXA1	TGCGCCACAAGCAAACCAGC	TCCAGGATGGCTTGGCAAAGGG
ARHGAP25	CTGAGAGACGCTTTTGATGCT	TCTCGGAGGTAGAGCTTTAACA
ASPHD2	GCGTTCCATGAAGGTTCAGC	TGAAATCAAGAGCCTGCCGT
AZU1	CGGCGTGGCCTCCTTTTCCC	CCCCGGTCCCGGGTTGTTGA
B3GNT2	CGGGCTAATGACCGAGAATAC	CTGCTCAAAGAGGTTGACAGG
BCL2A1	GGTCACGTCGGTGGTTACGGG	TGGCCCCAGGATTCCCGGAT
BOC	CTCTGCAGTGGCAGGCGAGG	CCGTGCCCCTTGCTGTTCCC
BRWD1	CCAGCGCATCGGTCCTATG	CTTCCTGCACCAAGTAAAGAAGT
C12orf35	CCAAAGAGTGCTACATTACCACC	TGGTTACTTCCAGGATAGCTGAA
C14orf149	GAGGCCCGCGTCAATATCC	CCATCACCTTTCCATGTCCAG
C1orf38	ACCTTGGTGACAGCAGCGCA	GGGTGAGAGTGGGGGTGGCA
C20orf103	GCTCCCTGCAGCAAGACCCC	TGCTGCAGTCGCTGGATGCG
CASP10	TAGGATTGGTCCCCAACAAGA	GAGAAACCCTTTGTCGGGTGG
CASP4	TCCAGCATCCTTGGAAGTGGCCT	CCAGGACACGTTGTGTGGCGT
CCDC146	ACGCCTTGGCCATCGCTGAAA	TCGGACGCTGCTCTGCAGTT
CD164	TGGGGAAAGGTCGGTTTACCTCACA	TGCCACCTTGGGAGGAATGGA
CD36	ACTCAGTGTTGGTGTGGTGA	TGTGCTTGAGCCAGGTTTAT
CFLAR	AGAGTGAGGCGATTTGACCTG	GTCCGAAACAAGGTGAGGGTT
CHI3L2	GTCTCTCTGGGCAGGTGTAGTGGTC	TTCCTGGTTCCTGCCGGTCCT
CKB	GCTGACCGCCTGGGCTTCTC	GGGCAGGCATGAGGTCGTCG
CMPK2	GTACCTCCTTTATTCCTGAAGCC	ATGGCAACAACCTGGAACTTT
CXorf21	TGCAGGAGGGGTTAGTGAAGGGG	GCTTCCTTGCTGTACACACCTCTC
DCLRE1C	AGCACAAATGCAGATTCCCAGAGC	TCTCACCAGTTGCCAGCTTCTCA
DDAH2	GGACACGGCCCTAATCACG	CGTTCTCGTCTCCTATTTCCAC
DDX60L	TGCCAAAAGCTGGGTATTCCA	GTGAATTGTCCTCCGTTACTCAG
ECE1	TGTCCTAGGGCTGGGGGTGC	TAGTGGCAGTGGCAGGGGCT
EGR1	GCCTGCGACATCTGTGGAA	CGCAAGTGGATCTTGGTATGC
ENDOD1	GAAGCAAGCAAGCCTTGAATAC	AGGGAGAATGGGTAAAGCTGT
ENTPD1	GCCTCAGCAACTACCCCTTTG	GTAATCCAGCCATAGGCACCT
ETV4	AGAGCTTTAAGCAAGAATACCATGATC	GGGTACCTGTGCCCATTGAC
EVL	TCCTCAGAGGCTGGCCGGAA	GCTTCGGGGCTCTTTGCCACA
FAM109B	ACATGCGCCTGGTGGTACGC	GCCCGCAGCTCGGTGGTTAG
FAM122C	ACGGGAGCCGGAAAGCCTTG	CGGTAGTGGAACTCGGCAGCG

Table 1 qPCR primers (Continued…)

Gene Symbol	Forward Primer(5'-3')	Reverse Primer(5'-3')
FAM127C	TCCTGGCAGCCAAATCGAG	AGCAGAATGCAGGGGTTACT
FCGR2C	TCATTGTGGCTGTGGTCACTGGG	TTGGGCAGCCTTCACAGGATCAG
FGD2	AGCTAACCCCCGCATCGGTGA	CAGCTCAGCCGCTCGCTCAA
FNDC5	GCATTCTAGAGGGCAGAGAAATTC	GTCATAAGGGTCAAAGAAGATAGAGGAT
FOSB	GCCAATGCTCCAGCTGTCGTCT	TGTGGTTGGCAGGAGCAAGCC
FRMD3	GGTGATTACGATCCTGATGAGC	CCACCCCGTAGGTTTCCAAAG
FRY	AGGACGGTGCCCGAGAGCAG	GAGAACGTCTGCGCACTCCCC
FTSJD2	TGCCCCGGGACACTCTGCTA	TCCCGAACGTCGGTGCCATT
GALM	GGGAAGGAGTATCACCTGGC	AGACTTTTAACTCTCCGGGGTAG
GAPDH	TCGGAGTCAACGGATTTG	GCATCGCCCCACTTGATT
GLYATL1	AGTCCCGGTCTCATGGGTAA	AGAAACCAAACTGCCCCACA
GPR84	TTGGCATCTTCTATTGCCTCATC	TGTCGCAACTTGTATTGGTCC
GSDMD	GTGTGTCAACCTGTCTATCAAGG	CATGGCATCGTAGAAGTGGAAG
GSN	GGTGTGGCATCAGGATTCAAG	TTTCATACCGATTGCTGTTGGA
IDH1	GCCCACGGGACTGTAACCCG	GCTCTGTGGGCTAACCCTCTGGT
IER3	GGTGCGCGAGAGCGTATCCC	TCCTTCCCACCGGGCCTAGC
IFI6	CAGAAGGCGGTATCGCTTTTC	CCTGCATCCTTACCCGCATT
IL4I1	ACTCGCCCGAAGACATCTAC	CATCCTCGGACATCACGTCTC
INPP1	ACCTTGAGGTTGTGTTCAACAG	GAGGACTTTGCTAAGAAGCTCTG
ITGB7	TGCGGAGGCTTTGGTCGCTG	ACTGCAGAGCCCTCCCTCGG
KIAA0226	GGAAGCGCTGGCCCTGGAAG	CAACAGGTGTGACCCGGCCC
LHFPL1	GCTGTGCCTTATACCCTTTAGG	GTTTGCATTATCTCCGGGCTAT
LHFPL2	AGAGATGGAATGGGACAACCGCA	ACCTCCCACCCCTTTTGCCCT
LILRA6	TCCAACCCCCACCTGCTGTCTT	ATCCTTGGCGTGTGAGGCAGGT
LOC100132707	CTCAGCCCCGACACACAGGC	GGCCTCAGTTCTGGCCGCAT
LOC285033	TGTGAGACTGTTCCTCGTAGC	AGAGTTGACCCGAGATGTCTT
LOC340515	AGCTGGCTTACCAGAGCACCGA	CCCAGCGCTGTAAGGAGTCGC
LPIN2	TCTACAAGGGCATTAACCAGGC	TGCTGTACCACGATGACATCA
MACROD1	GTGTTTGGCTACCCCTGTGA	CTCGAGGAACACGCAGATGA
MC3R	GCGACTACCTGACCTTCGAG	TAGCGGAGCGCGTAAAAGAT
MFN1	ACCCATGCCAAGGAGCGAGC	GGCACAGGCGAGCAAAAGTGG
MGC87042	TGTGGGACTGGCAATACTGGCTCT	TGCTGTGCTCCAAAGCCTCGTT
MIR155HG	AGCAAGCGCGGGGAACCAAG	CCCCTTCCTGGTTTGTGCCACC
MOXD1	GAAGCTGGTCCCAAGTACCAT	AGGATTCAATAACCGCAAACTCT
MR1	GCACTGCGGTGTCCACATGGT	GCTCTCGGGGCCTTCTTCTCCA

Table 1 qPCR primers (Continued…)

Gene Symbol	Forward Primer(5'-3')	Reverse Primer(5'-3')
MS4A12	TGGCCAAGACTACTGGGCCGT	TGGGCTGTGGCACAAGCTACG
MS4A13	ACAACCTGCCCCAGAAGAAT	CCTGGTTGGGAGACTAAAGGAC
MSMB	GCACCCTTGTTTCTACACCTGTGGG	TGGGTCCTTCTTCTCCACCACGA
MST1R	CTTTGACGTGAAGTACGTGGT	CGTATGGCTACAAACACAGCAC
MVP	TACATCCGGCAGGACAATGAG	CTGTGCAGTAGTGACGTGGG
MYO5C	GGATGGAACGGAGCTGGATT	AGCGGATTCTGAGGTTGTGG
N4BP2L2	ACTTCGTGCTCTTTGGGAGTCAC	AGTCTTGGCTGTCCTCAGCTGGT
NAPSA	AATGGCGTCCGCCTCTGCTT	GGCGCTGCTCTTCATGTCCCC
NEXN	GCTCCCCTCCCCTCTCCCTG	TTGGCCCCGCCACAGAAAAGA
NFKBIE	GCCTTGCTGGGTGGGGTCAG	AACCCCCAGGGCAGTGGTGA
NPC2	TGACAGCACCTCCAGCTCTGCT	TGAGAACCAGCCACCCGGAGC
NPHP1	ATCGCTGTTGGAGATTTTACTGC	GGCTCTAGGTAGGTTCTGGGA
NUB1	AGGATTCAACTTTGGAAACCTCC	ACAGCATTCTAGTCTGTCAGAGT
PARP3	TGGCCAGCAAGTGGTGGTGC	GGTAGCGCAGGCGACACTGG
PATL2	CAGCCTCTCTGGCAGAACCCC	CTGGCCTCCAGCTGCTGAACC
PDE6H	CCAGGGTCCTACCACCCCACG	TGGCGAGTCTGCCTCTGCTTG
PDS5A	TTGCCTGACCCCGGGAAAGC	GCAAGTTTCCGGGCTATTTCTCTCA
PECAM1	AACAGTGTTGACATGAAGAGCC	TGTAAAACAGCACGTCATCCTT
PHLDA2	GGAATGGGCGGGCAGGACAC	CCGGTTCCCAGCGCCTTTCA
PIM1	GCGGCTTCGGCTCGGTCTAC	GGCAGCTCTCCCCAGTCGGA
PLAC8	TTCCCGTGCCTTGGGTGTCA	TTGCGACGCTTGTTCCACACA
PLEKHG7	TTAACTCAGACAAGCCTTGGTTT	TCTCAGAAGCGTCTACATAGTCC
PTPRO	ACAAGGGCTGTATACGGGGTTACAA	GGGCTCACATAATGGGGGAGACCA
RASGRP3	GGGAAAAGCCTGTCTGCTGTT	GCTCCAGAAAAGTGAGGTGCT
RB1CC1	ATCGAAGAGTGTGTACCTACAGT	GCAGGTGGACGATCACATAAGAT
REC8	GTGCTGGTACGAGTGCAAC	ATAGACGCGGATCACACCGA
RERE	ACAGGCCCTCATGGCAGGCT	GAAGCCGAGAAGGTGGGGGC
RNASET2	CCAGTGCCTTCCACCAAGCCAG	CCCCCGGCTCGGTGCAGTTT
RNF149	GGGTCAAAGGAGCAGGCTAATGC	TGCCTCATACTCGACTTCTACCTCC
RPS6KC1	TGGTGCTGGAGTTGCTGGTGTT	GCCTCAGTGCTGGAGATGCCT
RSC1A1	ACCATAGCTGAGGGCCAAACCA	TGGCCTGATGAAAGGAGACTTGTGG
RUNX3	GCACCCAAGGGGCCTCTCCA	TCCCACGCTGACCTGGGACC
SECISBP2L	CCAACTCCAATTCCCAGCTAC	GGGTTGTTGCCATCGTATATCA
SELL	ACCCAGAGGGACTTATGGAAC	GCAGAATCTTCTAGCCCTTTGC
SEMA4G	CACCGGGGCACTTTCAGGGG	TCACCCGAGGGGAATGGGGG

Table 1 qPCR primers (Continued…)

Gene Symbol	Forward Primer(5'-3')	Reverse Primer(5'-3')
SEMA7A	TCCCCAGGGGCTGTGCTTCC	GAGAGCTCGGCCCAGGCCAT
SH3BP2	TCCCGCACCTCCAGCGATCA	CCCGCCAGTGTCAGCCTGTG
SHROOM3	GGACCTGCTGGAACGCTCGG	AGCACATCCTGGCGCTTGTCT
SIGLEC14	AGCCCTGCGGATCCTGAGCAA	TGCAGGCGAGGAACAGGGACT
SLC24A1	CCATTGCGGTGGATGAGCTA	CTGTTGTGCAGAGAGGTCG
SLC38A5	GGCCTTCAGCTGGCCACGAC	GGGGCTGAGGTGGACCCGATAA
SNX9	ATGGCCCAATGTGGGTTTATC	AGGAGACGCTCATATAACCAGTC
SP140	AGGATGGTCGCAGAGATCCA	TGGCCTTGTTATTGCACTTGC
SP140L	AACCTGGAACCCACTTTACTCA	TCTTGCGTAGCCTCCTTTGAT
ST3GAL5	CAGAGGGCGCACCACTGTCTG	AGAGTCGTACCCAGAATGGCAGGG
STON2	AGCAGTCCTAGCTTTGGATGT	AATTGTCAGTTGAGTCAGCCC
TEX14	AGTCCCCTGTCCTGTTCAACT	GCCGCAACAAAAAGTGCTGT
TIMD4	GAAAGACCAGTGCCCCTACTC	ACCTCTCGGGATAGTCCCCT
TIPARP	ATGGCAGGGATTGTTTGAAGC	GCCACTTTTGAGTAGTTGTCCTT
TKTL1	TGGACAATCTTGTGGCAATCTT	CAGCGCCTCTGATAGATGTTTAT
TMEM106A	GCTTTTGCACATCCAGGGCACC	CTGGTGGGGCACAGATGCGT
TMEM217	AGGTCAGAATCATGCGCTGG	CCTGGCTCCGGTTTTTGTAGG
TMEM229B	CAGAGCCCCCTAACGTCCGC	CCAACCCCTGAACTGGGCCG
TMEM45B	GCTTCCAGGGAGTTTCTTCCT	CTTCCGCGTGTGGCTAAAGTA
TNFRSF1A	TCCGACCCCATCCCCAACCC	GGGGCACGTTCTCCACCACG
TOR1B	GCACCCCGGGATCATTGACGC	GTCCCCGCCTGCATTGCTGA
TPPP2	AAGTCAAGGCCAAGAACGCCCGA	TGGCTGGGTCTTTGCCCTCCA
TRANK1	TCCAACCTACGTGAAGGGATAC	GTGCCTGGTCTTGGCTTCTC
TRIM31	AGAGCTGGGGCTTGTTACAGA	GCCAGGAGACATACTGGAAAAG
UTRN	GTTCCACCAGGCAGCGGGAG	TGGCCTGCTGGGAACATTTGGG
WDFY1	ACCGACCGCATTGTAAAGATCTGGG	CGCTGTGGAGCTGCGTGAGA
XAF1	GCAGGTTGGGTGTACGATGT	GCTGCCACAGTAGGACTCG
XRN1	GAGAAGCGATTATTGGAAGCCA	GCACATTAGGCACTCACTATGTT
XRN2	GAGCCCAGCCTAACCATGAC	TGTGGCAAGGCCAAGCATAA
ZC3H12D	TGGCAGCATTGTCCTTATGGCAAGA	CGGCCTCTCCGGGTGGTAGA
